# Allelic loss of 10q23, the *PTEN* tumour suppressor gene locus, in Barrett's oesophagus-associated adenocarcinoma

**DOI:** 10.1054/bjoc.2000.1660

**Published:** 2001-03

**Authors:** M H Kulke, R D Odze, K S Thakore, G Thomas, H Wang, M Loda, C Eng

**Affiliations:** Department of Adult Oncology, Dana-Farber Cancer Institute and Harvard Medical School, Boston, MA; Department of Pathology, Brigham and Women's Hospital and Harvard Medical School, Boston, MA; Department of Pathology, Beth-Israel-Deaconess Medical Center and Harvard Medical School, Boston, MA; Human Cancer Genetics Program, Ohio State University, Columbus, Ohio and CRC Human Cancer Genetics Research Group, University of Cambridge, Cambridge, UK

**Keywords:** *PTEN*, Barrett's oesophagus, oesophageal adenocarcinoma, 10q23

## Abstract

*PTEN* is a putative tumour suppressor gene located on chromosome band 10q23. Mutations in *PTEN* have been identified in numerous human malignancies, including cancers of the brain, endometrium, ovary, and prostate. In this study, we screened 80 Barrett's oesophagus-associated adenocarcinomas (BOAd) for loss of heterozygosity (LOH) at 10q23, using the microsatellite markers D10S541, D10S219, and D10S551. Tumours demonstrating LOH were then screened for the presence or absence of *PTEN* mutations. LOH at one or more loci was identified in 17/80 (21%) cases. In none of these cases did we detect mutations in *PTEN*. The presence of LOH did not correlate with patient age, tumour stage, degree of differentiation, presence of perineural or vascular invasion, or overall survival. We conclude that LOH at chromosome 10q23 is uncommon in BOAd, is not associated with mutations in the *PTEN* tumour suppressor gene, and does not correlate with the clinical or pathologic features of these tumours. It is possible that *PTEN* is inactivated through other mechanisms in BOAd. © 2001 Cancer Research Campaign http://www.bjcancer.com


*British Journal of Cancer* (2001) **84**(6), 748–753
© 2001 Cancer Research Campaign
doi: 10.1054/ bjoc.2000.1660, available online at http://www.idealibrary.com on IDEAL®

### Allelic loss of 10q23, the *PTEN* tumour suppressor gene locus, in Barrett's oesophagus-associated adenocarcinoma

**MH Kulke[1], RD Odze[2], KS Thakore[1], G Thomas[1], H Wang[3], M Loda[1,2] and C Eng[4]**

[1]Department of Adult Oncology, Dana-Farber Cancer Institute and Harvard Medical School, Boston MA; [2]Department of Pathology, Brigham and Women's Hospital, and Harvard Medical School, Boston MA; [3]Department of Pathology, Beth-Israel-Deaconess Medical Center and Harvard Medical School, Boston MA; [4]Human Cancer Genetics Program, Ohio State University, Columbus, Ohio, and CRC Human Cancer Genetics Research Group, University of Cambridge, Cambridge, UK

**Summary** *PTEN* is a putative tumour suppressor gene located on chromosome band 10q23. Mutations in *PTEN* have been identified in numerous human malignancies, including cancers of the brain, endometrium, ovary, and prostate. In this study, we screened 80 Barrett's oesophagus-associated adenocarcinomas (BOAd) for loss of heterozygosity (LOH) at 10q23, using the microsatellite markers D10S541, D10S219, and D10S551. Tumours demonstrating LOH were then screened for the presence or absence of *PTEN* mutations. LOH at one or more loci was identified in 17/80 (21%) cases. In none of these cases did we detect mutations in *PTEN*. The presence of LOH did not correlate with patient age, tumour stage, degree of differentiation, presence of perineural or vascular invasion, or overall survival. We conclude that LOH at chromosome 10q23 is uncommon in BOAd, is not associated with mutations in the *PTEN* tumour suppressor gene, and does not correlate with the clinical or pathologic features of these tumours. It is possible that *PTEN* is inactivated through other mechanisms in BOAd. © 2001 Cancer Research Campaign http://www.bjcancer.com

**Keywords**: *PTEN*; Barrett's oesophagus; oesophageal adenocarcinoma; 10q23

In North America, the incidence of oesophageal adenocarcinoma is increasing at a rate higher than any other malignancy (Blot et al, 1991). The development of oesophageal adenocarcinoma is related to chronic gastro-oesophageal reflux and the subsequent development of Barrett's oesophagus (Lagergren et al, 1999). However, the molecular events leading to the development of oesophageal adenocarcinoma remain poorly understood.

Loss of tumour suppressor gene function may play a role in the development of oesophageal adenocarcinoma. Allelotype analysis of oesophageal adenocarcinoma specimens has revealed frequent loss of heterozygosity (LOH) at several sites of known tumour suppressor genes. These sites include 17p (*p53*), 18q (*DCC*); 9p21 (*CDKN2/p16*), and 5q (*APC*) (Huang et al, 1992; Hammoud et al, 1996; Dolan et al, 1998). *PTEN* has recently been identified as a novel tumour suppressor gene that is deleted or mutated in a wide range of human malignancies (Li et al, 1997; Steck et al, 1997). *PTEN* is located on chromosome band 10q23, and encodes a 403 amino acid dual specificity phosphatase that contains regions of homology to tensin and auxillin, cytoskeletal proteins that interact with adhesion molecules (Myers et al, 1997). Germline mutations of *PTEN* have been found in Cowden syndrome, an autosomal dominant inherited cancer syndrome characterized by hamartomas of the skin, intestine, breast and thyroid, and associated with a high risk of breast and thyroid cancers (Liaw et al, 1997). Germline mutations in *PTEN* have also been found in Bannayan-Zonana syndrome, which is characterized by intestinal hamartomatous polyps, lipomatosis, macrocephaly, and speckled penis, as well as in a Proteus-like syndrome (Marsh et al, 1997a, 1999; Zhou et al, 2000).

Somatic mutations of *PTEN* have been found in sporadic tumours of the breast (Teng et al, 1997; Chen et al, 1999; Freihoff et al, 1999), thyroid (Dahia et al, 1997), head and neck (Okami et al, 1998; Shao et al, 1998), central nervous system (Liu et al, 1997; Rasheed et al, 1997; Teng et al, 1997; Wang et al, 1997; Bostrom et al, 1998; Chiariello et al, 1998; Duerr et al, 1998; Maier et al, 1998; Davies et al, 1999; Zhou et al, 1999), endometrium (Kong et al, 1997; Risinger et al, 1997; Tashiro et al, 1997; Simpkins et al, 1998; Mutter et al, 2000; Yaginuma et al, 2000), ovary (Tashiro et al, 1997; Teng et al, 1997; Obata et al, 1998; Saito et al, 2000), prostate (Cairns et al, 1997; Dong et al, 1998; Gray et al, 1998; Pesche et al, 1998; Suzuki et al, 1998; Feilotter et al, 1999), kidney (Teng et al, 1997; Alimov et al, 1999), lung (Kohno et al, 1998; Yokomizo et al, 1998), and in melanomas (Teng et al, 1997; Tsao et al, 1998) and non-Hodgkins lymphomas (Gronbaek et al, 1998; Nakahara et al, 1998; Sakai et al, 1998; Butler et al, 1999; Dahia et al, 1999). Whether loss of *PTEN* function plays a role in the development of Barrett's oesophagus-associated adenocarcinoma (BOAd) is not known. In this study, we determined the prevalence and clinical significance of LOH at 10q23 in 80 cases of BOAd. Tumours demonstrating LOH were screened for *PTEN* mutations to determine if *PTEN* inactivation plays a role in the development of this type of malignancy.

*Received 17 July 2000*
*Revised 14 November 2000*
*Accepted 30 November 2000*

*Correspondence to:* MH Kulke

### MATERIALS AND METHODS

### Study group

80 patients who had en bloc oesophageal resection at the Brigham and Women's Hospital and at the Beth Israel-Deaconess Hospital between 1973 and 1995 were identified. All patients had histologically confirmed BOAd, and none had received preoperative chemotherapy or radiation. All patients were treated surgically with an intent to cure.

Selected clinical information (patient age, gender) and follow-up data were obtained from review of the patient's hospital charts and the hospital tumour registry, or from direct telephone interviews with the patient and/or his/her family when necessary. Follow-up time was calculated from the date of initial diagnosis to either the date of death or, for the patients who were still alive, to the date of the most recent clinical investigation. In the survival analysis, either death or tumour recurrence was considered a failure (event). Patients alive without disease at last follow-up were censored in the analysis.

### Pathologic analysis

All oesophageal resection specimens were received in the surgical pathology laboratory in the fresh state and fixed in 10% buffered formalin for subsequent tissue sectioning. Tissue sections were processed routinely, embedded in paraffin, and stained with haematoxylin and eosin (H & E).

The following microscopic features were evaluated in all cases by one of the authors (RDO): 1) Pathologic stage according to the 1993 revised AJCC TNM classification (Fleming et al, 1997); 2) The presence or absence of lymphovascular invasion; 3) The presence or absence of perineural invasion; 4) Degree of tumour differentiation (well, > 95% of the tumour composed of glands; moderate, 50–95% of the tumour composed of glands; poor, < 50% of the tumour composed of glands).

### Molecular analysis

Sections from paraffin-embedded tumour specimens were cut. Tumour and normal tissue were identified and separated by microdissection. DNA extraction was performed using the QIAprep kit (Qiagen Inc, Chatsworth, CA). PCR amplification was performed using primers for 3 known microsatellite repeat sequences: D10S219, D10S541, and D10S551. PCR primers were 5′ tagged with fluorescent dye labels. PCR products were then electrophoresed on 6% denaturing polyacrylamide gels and results were analysed using GeneScan 672 collection and analysis software (Genescan, Applied Biosystems, Foster City CA). Loss of one PTEN allele was established when the normal:tumour DNA peak ratio was greater than 1.5:1.

All cases demonstrating LOH were analysed further with denaturing-gradient gel electrophoresis (DGGE). In these cases, DGGE was completed for all nine exons of PTEN. GC-clamped primers for each exon have been previously described (Guldberg et al, 1997; Marsh et al, 1997a, 1998). PCR products were generated using the following conditions: a 'hot start' at 95˚C for 10 min; followed by 40 cycles of 94˚C for 1 min, annealing at 55˚C for 1 min, and extension at 72˚C for 1 min; followed by 72˚C for 10 min. Heteroduplexing of PCR products was performed with one cycle of 98˚C for 8 min, 55˚C for 30 min, and 40˚C for 30 min. PCR was performed in 1X PCR buffer (Life Technologies Inc.) 0.4 μM primer (Life Technologies, Inc. and 2.5 units of Taq polymerase (Life Technologies, Inc) with TaqStart antibody (Clontech, Palo Alto, CA). PCR products were separated on 1 mm 10% polyacrylamide gels with a gradient of 15–20% urea and 0–10% glycerol. Gels were run at 100 V for 16 h at 60˚C.

Cases in which the DGGE analysis was not definitive were sequenced directly. In these cases, the exon in question was sequenced using nested primers designed within the flanking intronic sequences. PCR conditions and primers for sequencing have been previously described (Liaw et al, 1997; Marsh et al, 1997b; Steck et al, 1997).

### Statistical analysis

The data analysis was done with STATA statistical software (STATA Corporation, College Station, Texas). Comparison of categorical data was done with either chi-square or Fisher's exact test, depending on sample size. Comparison for numeric data was done with the *t*-test. Survival analysis for clinical and pathologic variables was performed using a log-rank test. All variables that were statistically significant by univariate analysis ($P < 0.05$) were also evaluated by multivariate analysis. Kaplan–Meier curves were determined for selected groups of patients for comparison of survival.

### RESULTS

A total of 80 BOAd specimens were analysed. Of the 80 samples, 63 had pathologic and clinical follow-up data. The demographic and pathologic features of the patients are summarized in Table 1. Patients had a mean age of 62 years and were predominantly male (M:F = 8:1). The mean follow-up time was 33 months. At the time of last evaluation, 22 (35%) were alive and disease-free, 1 (2%) was alive with disease, and 39 (62%) had died of disease. All stages of disease were represented in the group: 12 (19%) patients had stage I, 14 (22%) stage IIA, 9 (14%) stage IIB, 24 (38%) stage III, and 4 (6%) stage IV lesions.

**Table 1** Demographic and pathologic features of the patient population

| Characteristic | | No. of patients | |
|---|---|---|---|
| Number of patients | | 63 | |
| Mean age (years) | | 62 | (37–87) |
| Male:female ratio | | 8:1 | (56:7) |
| Follow-up (months) | | 33 | (1–204) |
| Survival status | | | |
| | Alive without disease | 22 | (35%) |
| | Alive with disease | 1 | (2%) |
| | Dead of disease | 39 | (62%) |
| | Dead of other causes | 1 | (2%) |
| Pathologic stage | | | |
| | I | 12 | (19%) |
| | IIA | 14 | (22%) |
| | IIB | 9 | (14%) |
| | III | 24 | (38%) |
| | IV | 4 | (6%) |
| Tumour differentiation | | | |
| | Well-differentiated | 4 | (7%) |
| | Moderately differentiated | 32 | (55%) |
| | Poorly differentiated | 22 | (38%) |
| Perineural invasion | | 16 | (25%) |
| Vascular invasion | | 25 | (40%) |

© 2001 Cancer Research Campaign 

80 samples were analysed for evidence of LOH at 10q23. A panel of loci was initially evaluated in DNA extracted from the paraffin-embedded samples. We found that several of these markers, including AFMa086, a polymorphic marker within the PTEN gene, did not yield reproducible results in the archival specimens. 3 microsatellite loci (D10S219, D10S541 and D10S551) were selected for further study based on their reproducibility in our specimens and on their location flanking the *PTEN* tumour suppressor gene (Figure 1). Of the 3 markers, D10S541 is telomeric and in closest proximity to *PTEN* (< 0.3 cM) whereas D10S219 and D10S551 are centromeric and more distant from the gene (9 cM and 6 cM, respectively). A representative example of LOH is shown in Figure 2. All but one case were evaluable for LOH with at least one microsatellite marker (Figure 3). LOH was found most commonly at the D10S541 locus (11/70; 15.7%) and less commonly at D10S219 (4/74; 5.4%) and D10S551 (4/76; 5.2%). In almost all cases, LOH was found at only one of 3 loci examined. In 2 cases, LOH was noted at 2 loci. The first (#31) demonstrated LOH at both D10S219 and D10S551, and the second (#55) demonstrated LOH at D10S219 and D10S541. In no cases did all 3 loci demonstrate LOH. The total prevalence of LOH at one or more loci was 17/80 (21%).

DGGE was performed on all cases demonstrating LOH to determine if *PTEN* mutations were present in the remaining allele. All 9 exons of *PTEN* were analysed. In all but 9 cases, DGGE analysis demonstrated no evidence of *PTEN* mutations. In these 9 cases, the results of DGGE were not interpretable, and direct sequencing of the *PTEN* exon in question was performed. In none of these cases were mutations in *PTEN* found.

No relationship was found between the presence of LOH at 10q23 and patient age ($P$ = 0.98), degree of tumour differentiation ($P$ = 0.58), tumour stage ($P$ = 0.43), presence of perineural invasion ($P$ = 0.32), or the presence of vascular invasion ($P$ = 0.37). There was no correlation between the presence of LOH and overall survival ($P$ = 0.63).

### DISCUSSION

An analysis of 80 cases of BOAd demonstrated LOH at chromosome 10q23 in 21% of cases. LOH was found most commonly with the microsatellite locus D10S541, which is only several hundred Kb from *PTEN*, and less commonly at the microsatellite

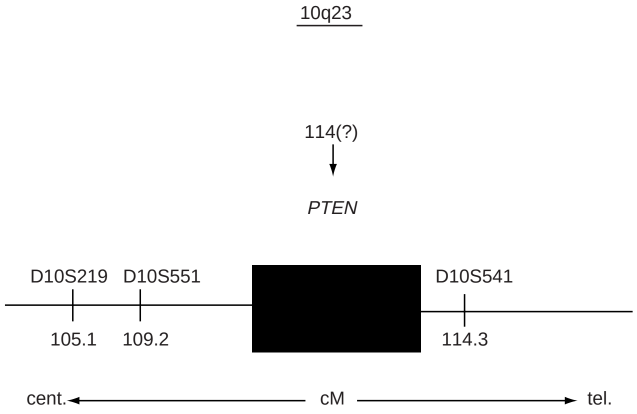

**Figure 1** Location of the microsatellite loci D10S219, D10S551, and D10S541 in relation to *PTEN*

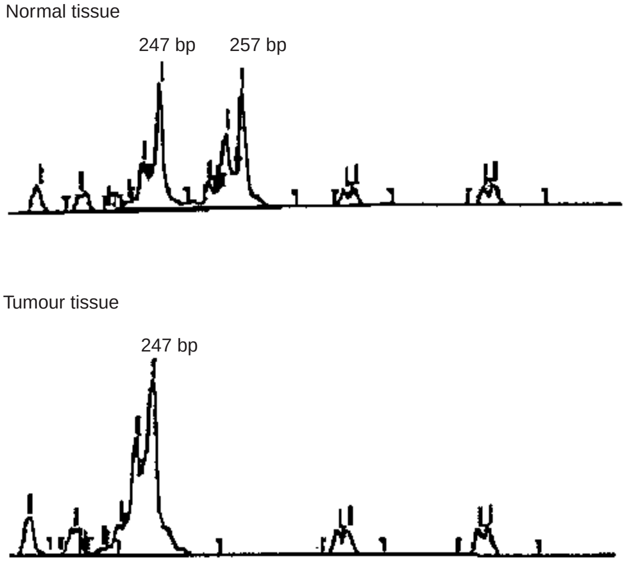

**Figure 2** Representative Genescan analysis demonstrating LOH at D10S541. Normal tissue demonstrates presence of two alleles, 247 and 257 bp in size. Tumour tissue demonstrates loss of the 257 bp allele

| Tumour # | D10S219 | D10S541 | D10S551 | Tumour # | D10S219 | D10S541 | D10S551 |
|---|---|---|---|---|---|---|---|
| 1 | | | | 41 | | | |
| 2 | | | | 42 | | LOH | |
| 3 | | | | 43 | | | LOH |
| 4 | | | | 44 | | | |
| 5 | | LOH | | 45 | | | |
| 6 | | | | 46 | | | |
| 7 | | | | 47 | | | |
| 8 | | | | 48 | | | |
| 9 | | LOH | | 49 | LOH | | |
| 10 | | | | 50 | | | |
| 11 | | | | 51 | | | |
| 12 | | | | 52 | | | |
| 13 | | | | 53 | | | |
| 14 | | | | 54 | | | |
| 15 | | | | 55 | LOH | LOH | |
| 16 | | | | 56 | | | |
| 17 | | | | 57 | | LOH | |
| 18 | | LOH | | 58 | | | |
| 19 | | LOH | | 59 | | | |
| 20 | | | | 60 | | LOH | |
| 21 | | | | 61 | | | |
| 22 | | | | 62 | | | |
| 23 | | | | 63 | | | |
| 24 | | | | 64 | | | |
| 25 | | | | 65 | | | |
| 26 | | | LOH | 66 | | | |
| 27 | | | LOH | 67 | | | |
| 28 | | | | 68 | LOH | | |
| 29 | | | | 69 | | | |
| 30 | | | | 60 | | | |
| 31 | LOH | | LOH | 71 | | | |
| 32 | | | | 72 | | LOH | |
| 33 | | | | 73 | | LOH | |
| 34 | | | | 74 | | | |
| 35 | | | | 75 | | | |
| 36 | | LOH | | 76 | | | |
| 37 | | | | 77 | | | |
| 38 | | | | 78 | | | |
| 39 | | | | 79 | | | |
| 40 | | | | 80 | | | |

| | |
|---|---|
| | No loss of heterozygosity |
| LOH | Loss of heterozygosity |
| | Not evaluable for technical reasons |

**Figure 3** LOH analysis of oesophageal cancer specimens

*© 2001 Cancer Research Campaign*

markers D10S219 and D10S551, which are more distant from the *PTEN* locus. Studies of the 10q23 region in other malignancies have also noted a higher rate of LOH at the D10S541 locus, leading to speculation that loss of this marker correlates closely with loss of PTEN. In an analysis of sporadic breast cancers using 11 microsatellite markers, LOH at D10S541 was found more commonly than with any other marker, and occurred in 55% of cases (Singh et al, 1998a).

To assess if biallelic structural defects of *PTEN* play a role in the development of oesophageal adenocarcinoma, we screened all 17 cases that demonstrated LOH for *PTEN* mutations using DGGE and, when necessary, direct sequence analysis. In no case did we find evidence of *PTEN* mutations in the respective remaining allele. The finding of 10q23 LOH without associated *PTEN* mutations is not unprecedented. Both 10q23 LOH and somatic *PTEN* mutations have been demonstrated in endometrial carcinomas (Kong et al, 1997; Mutter et al, 2000), endometrioid ovarian carcinoma (Teng et al, 1997; Obata et al, 1998; Saito et al, 2000), and high-grade gliomas (Teng et al, 1997; Wang et al, 1997; Bostrom et al, 1998; Chiariello et al, 1998; Zhou et al, 1999). However, despite the presence of 10q23 LOH, somatic mutations of *PTEN* are either absent or exceedingly rare in primary cancers of the pancreas (Okami et al, 1998), kidney (Teng et al, 1997; Alimov et al, 1999), bladder (Cairns et al, 1998; Aveyard et al, 1999), prostate (Cairns et al, 1997; Feilotter et al, 1998; Pesche et al, 1998; Suzuki et al, 1998), breast (Feilotter et al, 1999; Freihoff et al, 1999), thyroid (Dahia et al, 1997), head and neck (Shao et al, 1998; Gasparotto et al, 1999; Okami et al, 1998), and lung (Okami et al, 1998; Petersen et al, 1998).

Several investigators have suggested that the lack of *PTEN* mutations in these malignancies can be explained by the presence of another tumour suppressor gene located at 10q23 (Bostrom et al, 1998; Feilotter et al, 1998; Butler et al, 1999; Saito et al, 2000). This view has been supported by the identification, in several tumour types, of areas of 10q23 deletion distinct from *PTEN* (Singh et al, 1998a; Yeh et al, 1999). The relatively small number of loci analysed, and the absence of an intragenic marker in our study, raise the possibility that our findings of LOH were related to deletion of another gene at 10q23. However, the close proximity of D10S541 to *PTEN* (< 0.3 cM) and the higher incidence of D10S541 LOH in our study make it less likely that our findings are due to deletion of another tumour suppressor gene at this locus. Indeed, a high incidence of LOH at D10S541 was noted during fine structure deletion mapping of 10q22–24 in follicular thyroid adenomas and follicular thyroid carcinomas. In this study, LOH at D10S541 appeared to correlate with deletions of the *PTEN* gene (Yeh et al, 1999).

Other investigators have proposed that *PTEN* undergoes mechanisms of inactivation other than structural alteration, e.g. somatic mutation. An analysis of prostate cancer xenografts demonstrated decreased levels of both *PTEN* mRNA and PTEN protein in the absence of *PTEN* gene mutations (Whang et al, 1998). In this study, treatment with the demethylating agent 5-azadeoxycytidine restored mRNA expression, suggesting that *PTEN* may undergo inactivation by promoter methylation. Similarly, an analysis of leukaemia and lymphoma cell lines demonstrated decreased levels of *PTEN* mRNA and PTEN protein, despite the fact that only a small minority of these samples contained *PTEN* mutations (Dahia et al, 1999). Interestingly, several additional cell lines in this study demonstrated decreased protein levels despite normal or high levels of mRNA, suggesting that *PTEN* may be inactivated by both transcriptional silencing and by disruption at the protein level. Recently, multiple non-genetic mechanisms of PTEN inactivation have been observed in primary carcinomas of the thyroid, endometrium, cervix, and in melanomas (Gimm et al, 2000; Kurose et al, 2000; Mutter et al, 2000; Zhou et al, 2000).

Analyses of other tumour suppressor genes in oesophageal adenocarcinoma have demonstrated a similar high prevalence of LOH with a corresponding low rate of mutations. The *p16* tumour suppressor gene, located on 9p21, encodes a cyclin-dependent kinase inhibitor. Allelic loss of 9p21 has been found in 26–89% of oesophageal adenocarcinomas; however, mutations in *p16* are rare (Zhou et al, 1994; Gonzalez et al, 1997; Muzeau et al, 1997). Similarly, allelic loss of 5q has been reported in up to 75% of oesophageal adenocarcinomas, yet mutations of *APC* have been demonstrated in less than 10% of cases (Boynton et al, 1992; Zhuang et al, 1996; Gonzalez et al, 1997). Many of these tumour suppressor genes, like *PTEN*, may be regulated by mechanisms other than intragenic mutation. In the case of *p16*, small homozygous microdeletions appear to be a major mechanism of inactivation, as does methylation of the *p16* promoter (Liggett and Sidransky, 1998). Loss of expression of *p27*, a cyclin-dependent kinase inhibitor, has also been demonstrated in a wide range of malignancies, including oesophageal adenocarcinoma (Esposito et al, 1997; Tan et al, 1997; Singh et al, 1998b; Yang et al, 1998). The expression of *p27* appears to be regulated by proteolytic degradation rather than by genetic mutation (Singh et al, 1998b).

LOH at 10q23 in our study did not correlate with any of the clinicopathologic features of the tumours analysed, nor did it correlate with overall survival. These findings contrast with those in breast cancer, where 10q23 LOH has been associated with adverse prognostic factors, including higher stage, higher tumour grade, and loss of oestrogen receptors (Bose et al, 1998; Garcia et al, 1999). In gliomas, the presence of *PTEN* mutations correlated with high tumour grade (Rasheed et al, 1997; Duerr et al, 1998; Davies et al, 1999; Zhou et al, 1999). However, among high-grade glioblastomas, in which the incidence of *PTEN* mutation is highest, *PTEN* mutations do not appear to influence overall survival (Zhou et al, 1999). Given that *PTEN* may undergo regulation through mechanisms other than somatic mutation, the level of *PTEN* expression in various malignancies may be a more useful prognostic marker than the presence of *PTEN* mutation. Indeed, in prostate cancer, loss of PTEN protein expression has been found to be associated with both a high Gleason score and advanced tumour stage, both markers of poor prognosis (McMenamin et al, 1999).

Of the other tumour suppressor genes lost or mutated in oesophageal adenocarcinoma, both *p27* and *p53* have been analysed with regard to their effect on prognosis. Loss of p27 expression occurs in approximately 80% of BOAd, and is predictive of a poor prognosis (Singh et al, 1998b). Allelic loss of 17p, the site of the *p53* oncogene, is common in oesophageal adenocarcinomas, and *p53* mutations have been demonstrated in approximately 50% of cases (Huang et al, 1992; Hamelin et al, 1994; Neshat et al, 1994; Gleeson et al, 1995; Hammoud et al, 1996; Schneider et al, 1996; Dolan et al, 1998). However, *p53* mutations do not have any prognostic significance in patients with these tumours (Flejou et al, 1994; Vijeyasingam et al, 1994).

In summary, we have demonstrated that, while LOH at 10q23 occurs in a subset of BOAd, intragenic mutations in the *PTEN* tumour suppressor gene do not play a significant role in the

© 2001 Cancer Research Campaign development of these lesions. Furthermore, LOH at 10q23 does not correlate with the major clinicopathologic features of these tumours. It is possible that *PTEN* activity is regulated by mechanisms other than intragenic mutation in BOAd.

### REFERENCES

Alimov A, Li C, Gizatullin R, Fredriksson V, Sundelin B, Klein G and Zabarovsky E (1999) Somatic mutation and homozygous deletion of PTEN/MMAC1 gene at 10q23 in renal cell carcinoma. *Anticancer Res* **19**: 3841–3846

Aveyard J, Skilleter A, Habuchi T and Knowles M (1999) Somatic mutations of PTEN in bladder carcinoma. *Br J Cancer* **80**: 904–908

Blot W, Devesa S, Kneller R and Fraumeni J (1991) Rising incidence of adenocarcinoma of the esophagus and gastric cardia. *JAMA* **265**: 1287–1289

Bose S, Wang S, Terry M, Hibshoosh H and Parsons R (1998) Allelic loss of chromosome 10q23 is associated with tumor progression in breast carcinomas. *Oncogene* **17**: 123–127

Bostrom J, Cobbers J, Wolter M, Tabatabai G, Weber R, Lichter P, Collins V and Reifenberger G (1998) Mutation of the PTEN (MMAC1) tumor suppressor gene in a subset of glioblastomas but not in meningiomas with loss of chromosome arm 10q. *Cancer Res* **58**: 29–33

Boynton R, Blount P, Yin J, Brown V, Huang Y, Tong Y, McDaniel T, Newkirk C, Resau J, Raskind W, Haggitt R, Reid B and Meltzer S (1992) Loss of heterozygosity involving the APC and MCC genetic loci occurs in the majority of human esophageal cancers. *Proc Natl Acad Sci USA* **89**: 3385–3388

Butler M, Wang S, Chaganti R, Parsons R and Dalla-Favera R (1999) Analysis of PTEN mutations and deletions in B-cell non-hodgkins lymphomas. *Genes Chromosomes Cancer* **24**: 322–327

Cairns P, Okami K, Halachmi S, Halachmi N, Esteller M, Herman J, Jen Isaacs W, Bova G and Sidransky D (1997) Frequent inactivation of PTEN/MMAC1 in primary prostate cancer. *Cancer Res*

Cairns P, Evron E, Okami K, Halamachi N, Esteller M, Herman J, Bose S, Wang S, Parsons R and Sidransky D (1998) Point mutation and homozygous deletion of PTEN/MMAC1 in primary bladder cancers. *Oncogene* **16**: 3215–3218

Chen S, Yu S, Tsai M, Yeh K, Wang J, Kao M, Shih M and Chang J (1999) Mutation analysis of the putative tumor suppression gene PTEN/MMAC1 in sporadic breast cancer. *Breast Cancer Res Treat* **55**: 85–89

Chiariello E, Roz L, Albarosa R, Magnani I and Finocchiaro G (1998) PTEN/MMAC1 mutations in primary glioblastomas and short-term cultures of malignant gliomas. *Oncogene* **16**: 541–545

Dahia P, Marsh D, Zheng Z, Zedenius J, Komminoth P, Frisk T, Wallin G, Parsons R, Longy M, Larsson C and Eng C (1997) Somatic deletions and mutations in the Cowden disease gene, PTEN, in sporadic thyroid tumors. *Cancer Res* **57**: 4710–4713

Dahia P, Aguiar R, Alberta J, Kum J, Caron S, Sill H, Marsh D, Ritz J, Freedman A, Stiles C and Eng C (1999) PTEN is inversely correlated with the cell survival factor Akt/PKB and is inactivated via multiple mechanisms in haematological malignancies. *Hum Mol Genet* **8**: 185–193

Davies M, Gibbs F, Halliwell N, Joyce K, Roebuck M, Rossi M, Salisbury J, Sibson D, Tacconi L and Walker C (1999) Mutation in the PTEN/MMAC1 gene in archival low grade and high grade gliomas. *Br J Cancer* **79**: 1542–1548

Dolan K, Grade J, Gosney J, Sissons M, Wright T, Kingsnorth A, Walker S, Sutton R, Meltzer S and Field J (1998) Allelotype analysis of oesophageal adenocarcinoma: loss of heterozygosity occurs at multiple sites. *Br J Cancer* **78**: 950–957

Dong J, Sipe T, Hyytinen E, Li C, Heise C, McClintlock D, Grant C, Chung L and Frierson H (1998) PTEN/MMAC1 is infrequently mutated in pT2 and pT3 carcinomas of the prostate. *Oncogene* **17**: 1979–1982

Duerr E, Rollbrocker B, Hayashi Y, Peters N, Meyer-Puttlitz M, Louis D, Schramm J, Wiestler O, Parsons R, Eng C and Deimling A (1998) PTEN mutations in gliomas and glioneuronal tumors. *Oncogene* **16**: 2259–2264

Esposito V, Baldi A, DeLuca A, Groger A, Loda M, Giordano G, Caputi M, Baldi F, Pagano M and Giordano A (1997) Prognostic role of the cyclin-dependent kinase inhibitor in non-small cell lung cancer. *Cancer Res* **57**: 3381–3385

Feilotter H, Nagai M, Boag A, Eng C and Mulligan L (1998) Analysis of PTEN and the 10q23 region in primary prostate carcinomas. *Oncogene* **16**: 1743–1748

Feilotter H, Coulon V, McVeigh J, Goag A, Dorion-Bonnet F, Duboue B, Latham W, Eng C, Mulligan L and Longy M (1999) Analysis of the 10q23 chromosomal region and the PTEN gene in human sporadic breast carcinoma. *Br J Cancer* **79**: 718–723

Flejou J, Paraf F and Potet F (1994) p53 protein expression in Barrett's adenocarcinoma: a frequent event with no prognostic significance. *Histopathology* **24**: 487–489

Fleming I, Cooper J and Henson D (1997) *AJCC manual for staging of cancer*, 5th ed. Berlin, New York: Wiley

Freihoff D, Kempe A, Beste B, Wappenschmidt B, Kreyer E, Hayashi Y, Meindl A, Krebs D, Wiestler O, Deimling A and Schmutzler R (1999) Exclusion of a major role for the PTEN tumour-suppressor gene in breast carcinomas. *Br J Cancer* **79**: 754–758

Garcia J, Silva J, Dominguez G, Gonzalez R, Navarro A, Carretero L, Provencio M, Espana P and Bonilla F (1999) Allelic loss of the PTEN region (10q23) in breast carcinomas of poor pathophenotype. *Breast Cancer Res Treat* **57**: 237–243

Gasparotto D, Vukosavljevic T, Piccinin S, Barzan L, Sulfaro S, Armellin M, Boiocchi M and Maestro R (1999) Loss of heterozygosity at 10q in tumors of the upper respiratory tract is associated with poor prognosis. *Int J Cancer* **84**: 432–436

Gimm O, Perren A, Weng L, Marsh D, Yeh J, Ziebold U, Gil E, Hinze R, Delbridge L, Lees J, Robinson B, Komminoth P, Dralle H and Eng C (2000) Differential nuclear and cytoplasmic expression of PTEN in normal thyroid tissue, and benign and malignant epithelial thyroid tumors. *Am J Pathol* **156**: 1693–1700

Gleeson C, Sloan J and McGuigan J (1995) Base transitions at CpG nucleotides in the p53 gene are common in esophageal adenocarcinoma. *Cancer Res* **55**: 3406–3411

Gonzalez M, Artimez M, Rodrigo L, Lopez-Larrea C, Menendez M, Alvarez V, Perez R, Fresno M, Perez M, Sampedro A and Coto E (1997) Mutation analysis of the p53, APC, and p16 genes in the Barrett's oesophagus, dysplasia, and adenocarcinoma. *J Clin Pathol* **50**: 212–217

Gray I, Stewart L, Phillips S, Hamilton J, Gray N, Watson G, Spurr N and Snary D (1998) Mutation and expression analysis of the putative prostate tumour-suppressor gene PTEN. *Br J Cancer* **78**: 1296–1300

Gronbaek Y, Zeuthen J, Guldberg P, Ralfkiaer E and Hou-Jensen K (1998) Alterations of the MMAC1/PTEN gene in lymphoid malignancies. *Blood* **91**: 4388–4390

Guldberg P, Straten P, Birck A, Ahrenkiel V, Kirkin A and Zeuthen J (1997) Disruption of the MMAC1/PTEN gene by deletion or mutation is a frequent even in malignant melanoma. *Cancer Res* **57**: 3660–3663

Hamelin R, Flejou J and Muzeau F (1994) TP53 gene mutations and p53 protein immunoreactivity in malignant and premalignant Barrett's esophagus. *Gastroenterology* **107**: 1012–1018

Hammoud Z, Kaleem Z, Cooper J, Sundaresan R, Patterson G and Goodfellow P (1996) Allelotype analysis of esophageal adenocarcinomas: evidence for involvement of sequences on the long arm of chromosome 4. *Cancer Res* **56**: 4499–4502

Huang Y, Boynton R, Blount P, Silverstein R, Yin J, Tong Y, McDaniel T, Newkirk C, Resau J, Sridhara R, Reid B and Meltzer S (1992) Loss of heterozygosity involves multiple tumor suppressor genes in human esophageal cancers. *Cancer Res* **52**: 6525–6530

Kohno T, Takahashi M, Manda R and Yokota J (1998) Inactivation of the PTEN/MMAC1/TEP1 gene in human lung cancers. *Genes Chrom Cancer* **22**: 152–156

Kong D, Suzuki A, Zou T, Sakurada A, Kemp L, Wakatsuki S, Yokoyama T, Yamakawa H, Furukawa T, Sato M, Ohuchi N, Sato S, Yin J, Wang S, Abraham J, Souza R, Smolinski K, Meltzer S and Horii A (1997) PTEN1 is frequently mutated in primary endometrial carcinomas. *Nature Genet* **17**: 143–144

Kurose K, Zhou X, Araki T and Eng C (2000) Biallelic inactivating mutations and an occult germline mutation of PTEN in primary cervical carcinomas. *Gene Chrom Cancer* **29**: 166–172

Lagergren J, Bergstrom R, Lindgren A and Nyren O (1999) Symptomatic gastroesophageal reflux as a risk factor for esophageal adenocarcinoma. *N Engl J Med* **340**: 825–831

Li J, Yen C, Liaw D, Podsypanina K, Bose S, Wang S, Puc J, Miliaresis C, Rodgers L, MCombie R, Bigner S, Giovanella B, Ittman M, Tycko B, Hibshoosh H, Wigler M and Parsons R (1997) PTEN, a putative protein tyrosine phosphatase gene mutated in human brain, breast and prostate cancer. *Science* **275**:

Liaw D, Marsh D, Li J, Dahia P, Wang S, Zheng Z, Bose S, Call K, Tsou H, Peacocke M, Eng C and Parsons R (1997) Germline mutations of the PTEN gene in Cowden disease, an inherited breast and thyroid cancer syndrome. *Nat Genet* **16**: 64–67

Liggett W and Sidransky D (1998) Role of the p16 tumor suppressor gene in cancer. *J Clin Oncol* **16**: 1197–1206

 © 2001 Cancer Research Campaign

Liu W, James D, Frederick L, Alderete B and Jenkins R (1997) PTEN/MMAC1 mutations and EGFR amplification in glioblastomas. *Cancer Res* **57**: 5254–5257

Maier D, Zhang Z, Taylor E, Hamou M, Gratzl O, Meir E, Scott R and Merlo A (1998) Somatic deletion mapping on chromosome 10 and sequence analysis of PTEN/MMAC1 point to the 10q25–26 region as the primary target in low-grade and high-grade gliomas. *Oncogene* **16**: 5551–5555

Marsh D, Dahia P, Zheng Z, Liaw D, Parsons R, Gorlin R and Eng C (1997a) Germline mutations in PTEN are present in Bannayan-Zonana syndrome. *Nature Genet* **16**: 333–334

Marsh D, Roth S, Lunetta K, Hemminki A, Dahia P, Sistonen P, Zheng Z, Caron S, Orsouw N.v, Bodmer W, Cottrell S, Dunlop M, Eccles D, Hodgson S, Jarvinen H, Kellokumpu I, Markie D, Neale K, Phillips R, Rozen P, Syngal S, Vijg J, Tomlinson I, Aaltonen L and Eng C (1997b) Exclusion of PTEN and 10q22–24 as the susceptibility locus for juvenile polyposis syndrome. *Cancer Res* **57**: 5017–5021

Marsh D, Dahia P, Caron S, Kum J, Fralyling I, Tomlinson I, Hughes K, Eeles R, Hodgson S, Murday V, Houlston R and Eng C (1998) Germline PTEN mutations in Cowden syndrome-like families. *J Med Genet* **35**: 881–885

Marsh D, Kum J, Lunetta K, Bennett M, Gorlin R, Ahmed S, Bodurtha J, Crowe C, Curtis M, Dasouki M, Dunn T, Feit H, Geraghty M, Graham J, Hodgson S, Hunter A, Korf B, Manchester D, Miesfeldt S, Murday V, Nathanson K, Parisi M, Pober B, Romano C, Tolmie J, Trembath R, Winter R, Zackai E, Zori R, Weng L, Dahia P and Eng C (1999) PTEN mutation spectrum and genotype-phenotype correlations in Bannayan-Riley-Ruvalcaba syndrome suggest a single entity with Cowden syndrome. *Hum Molec Genet* **8**: 1461–1472

McMenamin M, Soung P, Perera S, Kaplan I, Loda M and Sellers W (1999) Loss of PTEN expression in paraffin-embedded primary prostate cancer correlates with high Gleason score and advanced stage. *Cancer Res* **59**: 4291–4296

Mutter G, Lin M, Fitzgerald J, Kurn J, Baak J, Lees J, Weng L and Eng C (2000) Altered PTEN expression as a diagnostic marker for the earliest endometrial precancers. *J Natl Cancer Inst* **92**: 924–931

Muzeau F, Flejou J, Thomas T and Hamelin R (1997) Loss of heterozygosity on chromosome 9 and p16 (MTS1, CDKN2) gene mutations in esophageal cancers. *Int J Cancer* **72**: 27–30

Myers M, Stolarov J, Eng C, Li J, Wang S, Wigler M, Parsons R and Tonks N (1997) PTEN, the tumor suppressor from human chromosome 10q23, is a dual specificity phosphatase. *Proc Natl Acad Sci (USA)* **94**: 9052–9057

Nakahara Y, Nagai H, Kinoshita T, Uchida T, Hatano S, Murate T and Saito H (1998) Mutational analysis of the PTEN/MMAC1 gene in non-hodgkin's lymphoma. *Leukemia* **12**: 1277–1280

Neshat K, Sanchez C and Galipeau P (1994) p53 mutations in Barrett's adenocarcinoma and high grade dysplasia. *Gastroenterology* **106**: 1589–1595

Obata K, Morland S, Watson R, Hitchcock A, Chenevix-Trench G, Thomas E and Campbell I (1998) Frequent PTEN/MMAC mutations in endometrioid but not serous or mucinous epithelial ovarian tumors. *Cancer Res* 2095–2097

Okami K, Wu L, Riggins G, Cairns P, Goggins M, Evron E, Halachmi N, Ahrendt S, Reed A, Hilgers W, Kern S, Koch W, Sidransky D and Jen J (1998) Analysis of PTEN/MMAC1 alterations in aerodigestive tract tumors. *Cancer Res* 509–511

Pesche S, Latil A, Muzeau F, Cussenot O, Fournier G, Longy M, Eng C and Lidereau R (1998) PTEN/MMAC1/TEP1 involvement in primary prostate cancers. *Oncogene* **16**: 2879–2883

Petersen S, Rudolf J, Bockmuhl U, Gellert K, Wolf G, Dietel M and Petersen I (1998) Distinct regions of allelic imbalance on chromosome 10q22–q26 in squamous cell carcinomas of the lung

Rasheed B, Stenzel T, McLendon R, Parsons R, Friedman A, Friedman H, Bigner D and Bigner S (1997) PTEN gene mutations are seen in high grade but not in low grade gliomas. *Cancer Res* **57**: 4187–4190

Risinger J, Hayes A, Berchuk A and Barrett J (1997) PTEN/MMAC1 mutations in endometrial cancers. *Cancer Res* **57**: 4736–4738

Saito M, Okamoto A, Kohno T, Takakura S, Shinozaki H, Isonishi S, Yasuhara T, Yoshimura T, Ohtake Y, Ochiai K, Yokota J and Tanaka T (2000) Allelic imbalance and mutations of the PTEN gene in ovarian cancer. *Int J Cancer* **85**: 160–165

Sakai A, Thieblemont C, Wellmann A, Jaffe E and Raffeld M (1998) PTEN gene alterations in lymphoid neoplasms. *Blood* **92**: 3410–3415

Schneider P, Casson A, Levin B, Garewal H, Hoelscher A, Becker K, Dittler H, Cleary K, Troster M, Siewert J and Roth J (1996) Mutations of p53 in Barrett's esophagus and Barrett's cancer: a prospective study of ninety-eight cases. *J Thorac Cardiovasc Surg* **111**: 323–333

Shao X, Tandon R, Samara G, Kanki H, Yano H, Close L, Parsons R and Sato T (1998) Mutational analysis of the PTEN gene in head and neck squamous cell carcinoma. *Int J Cancer* **77**: 684–688

Simpkins S, Peiffer-Schneider S, Mutch D, Gersell D and Goodfellow P (1998) PTEN mutations in endometrial cancers with 10q LOH: additional evidence for the involvement of multiple tumor suppressors. *Gynecol Oncol* **71**: 391–395

Singh B, Ittmann M and Krolewski J (1998a) Sporadic breast cancers exhibit loss of heterozygosity on chromosome segment 10q23 close to the Cowden disease locus. *Genes Chromosomes Cancer* **21**: 166–171

Singh S, Lipman J, Goldman H, Ellis F, Aizenman L, Cangi M, Signoretti S, Chiaur D, Pagano M and Loda M (1998b) Loss or altered subcellular localization of p27 in Barrett's associated adenocarcinoma. *Cancer Res* **58**: 1730–1735

Steck P, Pershouse M, Jasser S, Yung W, Lin H, Ligon A, Langford L, Baumgard M, Hattier T, Davis T, Frye C, Hu R, Swedlund B, Teng D and Tavtigian S (1997) Identification of a candidate tumor suppressor gene, MMAC1, at chromosome 10q23.3 that is mutated in multiple advanced cancers. *Nature Genet* **15**: 356–362

Suzuki H, Freije D, Nusskern D, Okami K, Cairns P, Sidransky D, Isaacs W and bova G (1998) Interfocal heterogeneity of PTEN/MMAC1 gene alterations in multiple metastatic prostate cancer tissues. *Cancer Res* **58**: 204–209

Tan P, Cady B, Wanner M, Worland P, Cukor B, Magi-Galluzzi C, Lavin P, Draetta G, Pagano M and Loda M (1997) The cell cycle inhibitor p27 is an independent prognostic marker in small (T1a, b) invasive breast carcinomas. *Cancer Res* **57**: 1259–1263

Tashiro H, Blazes M, Wu R, Cho K, Bose S, Wang S, Li J, Parsons R and Ellenson L (1997) Mutations in PTEN are frequent in endometrial carcinoma but rare in other common gynecological malignancies. *Cancer Res* **57**: 3935–3940

Teng D, Hu R, Lin H, Davis T, Iliev D, Frye C, Swedlund B, Hansen K, Vinson V, Gumpper K, Ellis L, El-Naggar A, Frazier M, Jasser S, Langford L, Lee J, Mills G, Pershouse M, Pollack R, Tornos C, Troncoso P, Yung W, Fujii G, Berson A, Bookstein R, Bolen J, Tavtigian S and Steck P (1997) MMAC1/PTEN mutations in primary tumor specimens and cell lines. *Cancer Res* **57**: 5221–5225

Tsao H, Zhang X, Benoit E and Haluska F (1998) Identification of PTEN/MMAC1 alterations in uncultured melanomas and melanoma cell lines. *Oncogene* **16**: 3397–3402

Vijeyasingam R, Darnton S, Jenner K, Allen C, Billingham C and Matthews H (1994) Expression of p53 protein in oesophageal carcinoma: clinicopathological correlation and prognostic significance. *Br J Surg* **81**: 1623–1626

Wang S, Puc J, Li J, Bruce J, Cairns P, Sidransky D and Parsons R (1997) Somatic mutations of PTEN in glioblastoma multiforme. *Cancer Res* **57**: 4183–4186

Whang Y, Wu X, Suzuki H, Reiter R, Tran C, Vessalla R, Said J, Isaacs W and Sawyers C (1998) Inactivation of the tumor suppressor PTEN/MMAC1 in advanced human prostate cancer through loss of expression. *PNAS* **95**: 5246–5250

Yaginuma Y, Yamashita T, Ishiya T, Morizaki A, Katoh Y, Takahashi T, Hayashi H and Ishikawa M (2000) Abnormal structure and expression of PTEN/MMAC1 gene in human uterine cancers. *Molecular Caricinogenesis* **27**: 110–116

Yang R, Naitoh J, Murphy M, Wang H, Phillipson J, deKernion J, Loda M and Reiter R (1998) Low p27 expression predicts poor disease-free survival in patients with prostate cancer. *J Urol* **159**: 941–945

Yeh J, Marsh D, Zedenius J, Dwight T, Delbridge L, Robinson B and Eng C (1999) Fine-structure deletion mapping of 10q22–24 identifies regions of loss of heterozygosity and suggests that sporadic follicular thyroid adenomas and follicular thyroid carcinomas develop along distinct neoplastic pathways. *Genes Chromosomes Cancer*

Yokomizo A, Tindall D, Drabkin H, Gemmill R, Franklin W, Yang P, Sugio K, Smith D and Liu W (1998) PTEN/MMAC1 mutations identified in small cell, but not in non-small cell lung cancers. *Oncogene* **17**: 475–479

Zhou X, Tarmin L, Yin J, Jiang H, Suzuki H, Rhyu M, Abraham J and Meltzer S (1994) The MTS1 gene is frequently mutated in primary human esophageal tumors. *Oncogene* **9**: 3737–3741

Zhou X, Li Y, Hoang-Xuan K, Laurent-Puig P, Mokhtari K, Longy M, Sanson M, Delattre J, Thomas G and Hamelin R (1999) Mutational analysis of the PTEN gene in gliomas: molecular and pathological correlations. *Int J Cancer* **84**: 150–154

Zhou X, Gimm O, Hampel H, Niemann T, Walker M and Eng C (2000) Epigenetic PTEN silencing in malignant melanomas without PTEN mutation. *Am J Pathol* **157**: 1123–1128

Zhuang Z, Vortmeyer A, Mark E, Odze R, Emmert-Buck M, Merino M, Moon H, Liotta L and Duray P (1996) Barrett's esophagus: metaplastic cells with loss of heterozygosity at the APC gene locus are clonal precursors to invasive adenocarcinoma. *Cancer Res* **56**: 1961–1964

© 2001 Cancer Research Campaign 
